# From CO_2_ to Bioplastic – Coupling the Electrochemical CO_2_ Reduction with a Microbial Product Generation by Drop‐in Electrolysis

**DOI:** 10.1002/cssc.202001235

**Published:** 2020-07-29

**Authors:** Markus Stöckl, Svenja Harms, Ida Dinges, Steliyana Dimitrova, Dirk Holtmann

**Affiliations:** ^1^ Electrochemistry, Industrial Biotechnology DECHEMA Research Institute Theodor-Heuss-Allee 25 60486 Frankfurt am Main Germany; ^2^ Institute for Anorganic and Analytic Chemistry Goethe-Universität Frankfurt am Main Max-von-Laue-Straße 7 60438 Frankfurt am Main Germany; ^3^ Institute of Bioprocess Engineering and Pharmaceutical Technology University of Applied Sciences Mittelhessen Wiesenstrasse 14 35390 Giessen Germany

**Keywords:** electrochemical CO_2_ reduction, formate, microbial electrosynthesis, c*upriavidus necator*, polyhydroxybutyrate

## Abstract

CO_2_ has been electrochemically reduced to the intermediate formate, which was subsequently used as sole substrate for the production of the polymer polyhydroxybutyrate (PHB) by the microorganism *Cupriavidus necator*. Faradaic efficiencies (FE) up to 54 % have been reached with Sn‐based gas‐diffusion electrodes in physiological electrolyte. The formate containing electrolyte can be used directly as drop‐in solution in the following biological polymer production by resting cells. 56 mg PHB L^−1^ and a ratio of 34 % PHB per cell dry weight were achieved. The calculated overall FE for the process was as high as 4 %. The direct use of the electrolyte as drop‐in media in the bioconversion enables simplified processes with a minimum of intermediate purification effort. Thus, an optimal coupling between electrochemical and biotechnological processes can be realized.

## Graphical abstract

**Drop of a life**: This study presents the concept of a drop‐in electrolysis: CO_2_ has been electrochemically reduced to the intermediate formate and was subsequently used as sole substrate for the production of the polymer polyhydroxybutyrate (PHB) by the microorganism *Cupriavidus necator* without downstream processes to combine both conversions.

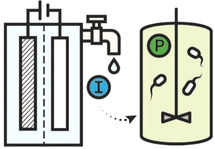

## Introduction

Driven by the scarcity of fossil raw materials and the increasing impacts of climate change on our lives, the chemical industry is faced with the great challenge of replacing fossil fuels with renewable alternatives to secure sustainable access to basic chemicals. Due to its key role as promotor of climate change via atmospheric accumulation, especially the greenhouse gas CO_2_ should be considered as a new raw material in the future.^1^, ^2^, ^3^, ^4^ Thereby, the focus lies on processes that enable the chemical activation of the thermodynamically stable CO_2_ using regenerative energy sources and convert it into storable and uncritical substances that can serve as starting materials for higher quality products. In this context, electrochemical processes offer the option to convert electricity originating from renewable energies directly into long term storable chemical energy.^5^ Water electrolysis is probably the most prominent and rather established example in recent literature.^6^, ^7^, ^8^, ^9^ However, various approaches for the electroreduction of CO_2_ are constantly emerging, with namely the electrosynthesis of syngas and formate as the two most promising options concerning short‐ to mid‐range industrial realization. The electrosynthesis of syngas from CO_2_ has already been carried out with superior Faradaic efficiencies (FE) at technical current densities over almost a year, demonstrating its industrial relevance and technical feasibility.^10^ However, as gaseous and toxic intermediate, the usage of syngas as well as H_2_ causes several challenges. Especially in terms of storage, safety issues due to the explosiveness and a low solubility in water both don't qualify as broad applicable alternatives for fossil raw materials. In contrast to syngas and H_2_, formate can be classified as mostly uncritical as well as easily storable electrochemical intermediate for carbon and energy, which is completely soluble in water.^11^, ^12^

Circumventing the low solubility of CO_2_ in aqueous electrolytes, the development and application of gas diffusion electrodes (GDE) is an important step towards industrially relevant electrolysis parameters for formate synthesis. Thereby, Sn was applied as main electro‐catalyst in the GDE.^13^, ^14^, ^15^, ^16^, ^17^, ^18^ In batch processes, FE up to 90 % have been reported at current densities in range of −50 to −200 mA cm^−2^.^13^ In continuous processes, several studies demonstrated FE between 70 und 75 % at current densities as high as −100 to −400 mA cm^−2^.^15^, ^16^, ^17^

**Scheme 1:**
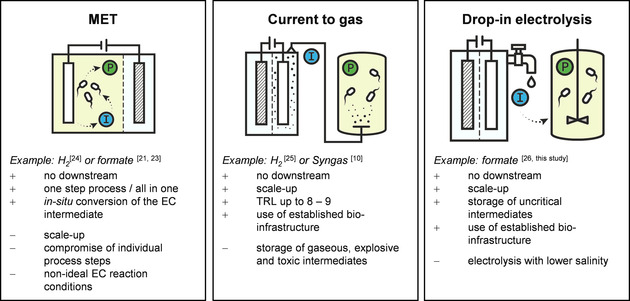
Schematic comparison of three different approaches for the conversion of current and CO_2_ to bio‐based products. Abbreviations: MET: secondary microbial electrochemical technologies; I: electrochemical intermediate; P: bio‐product; TRL: technical readiness level.

On the one hand side, electrochemical formate synthesis is a rather far developed technology for the storage of energy and CO_2_ binding compared to alternative electrochemical CO_2_ reduction routes besides syngas. On the other hand side, formate also offers great advantages as sustainable microbial feedstock for biotechnological transformation processes and the generation of higher value products, since it can be used as sole carbon source and serve as electron carrier in microbial electrosynthesis processes. As a natural fomatotroph *Cupriavidus necator* is an ideal candidate for the biotransformation of formate, nevertheless, there is still a great demand for more efficient and versatile pathways based on formate as sole carbon and energy source.^12^, ^19^ When combining electroreduction to form intermediates with bioproduction, different approaches are feasible (Scheme 1). One promising option are secondary microbial electrochemical technologies (MET), locating the initial electrochemical reduction in‐situ to the microbial biosynthesis,^20^, ^21^ as it has already been shown for formate^22^, ^23^ and H_2_.^24^ However, scale‐up issues and non‐ideal conditions of the electrochemical set up pose major challenges for establishing *in‐situ* production with direct bioconversion in industrial scale. This also applies to biofilm associated electrochemical processes, which are limited in terms of current density and mass transport due to the diffusion barrier biofilm. Consequently, a spatial separation of both the synthesis of the electrochemical intermediate and the subsequent biotransformation appears beneficial. Exemplarily, industrial solutions using H_2_ from electrolysis for the microbial upgrade of CO_2_ to methane are already commercially available.^25^ Furthermore, the bioconversion of syngas originating from electrolysis to alcohols has been reported with superior performance.^10^ Nevertheless, disadvantages of such gaseous electrochemical intermediates such as storage and toxicity as well as the limited applicability must be addressed. In production of soluble electrochemical intermediates such as formate, the separation of the intermediate from the electrolyte adds on as an additional process step (downstream processing).

Addressing these subjects, this study was aiming towards the scalable coupling of electrochemical formate synthesis and production of a bioproduct by a drop‐in electrolysis without an additional purification step. Recently, Chatzipanagiotou et al. =reported the electroreduction of CO_2_ to formate at copper (CuO_*x*_) electrodes in growth media and the subsequent transformation of formate by a mixed culture to methane and acetate.^26^ Reported current densities were around −2 mA cm^−2^, with electron recovery rates between 1 and almost 4 % for formate reduction in growth medium. For microbial growth and product generation, H_2_ and CO_2_ were additionally provided as energy carbon sources, respectively.

Inspired by the comparable process reported for syngas,^10^ in our study, formate was produced as electrochemical intermediate in a physiological electrolysis buffer to avoid downstream processing from CO_2_ reduction to bioconversion. Afterwards, the formate was used to produce polyhydroxybutyrate (PHB) with *Cupriavidus necator* (*C. necator*), demonstrating the scalable conversion of CO_2_ to a complex model product.

## Results and Discussion

### CO_2_ electrolysis to formate

**Figure 1:**
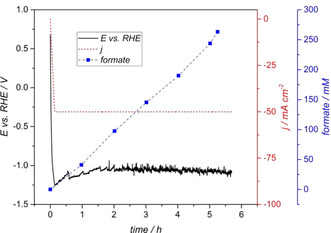
Time course of electrode potential (*E* vs. RHE, no iR‐drop correction), current density (*j*) and formate concentration during CO_2_ electrolysis at Sn‐based GDE in a custom designed electrolysis reactor. *n*=1.

The electrochemical reduction of CO_2_ to formate is exemplarily shown in Figure 1. The initial current increase (1 mA s^−1^) to the final current density of −50 mA cm^−2^, lead to a decrease of the electrode potential to a value of −1.26 V vs. RHE. At the constant current density the electrode potential decreased slightly and a mean value around −1.06 V vs. RHE was reached after approx. 2 h and remained relatively stable until the end of the electrolysis. The potential's decrease was mainly attributed to the depolarisation of the electrode, which is supposed to be smaller with slower increasing current rate at the beginning of the electrolysis. The noisy pattern of the potential signal was related to gas evolution at the GDE. The concentration pattern of the desired electrolysis product formate can also be seen in Figure 1. Formate was synthesized with a constant production rate of 50 mmol L^−1^ h^−1^, to a final concentration of 263.1 mm after 5.25 h with a Faradaic efficiency of 54 %. In order to produce a sufficient amount of formate containing buffer, three (*n*=3) electrolyses were run for 2 h each, resulting in 93.5±2.0 mm slightly lower FE at 51±1 %. This value was also used to calculate the overall Faradaic efficiency of the process. Current and potential curves are provided in the Supporting Information (Figure S1 in the Supporting Information). The average formate production rate was 47±1 mmol L^−1^ h^−1^ (*n*=3) and the pH value at the end of the electrolyses was 7.0±0.1 (*n*=3). Hydrogen evolution was observed during all electrolyses and is assumed to be the main unfavourable side reaction limiting the current efficiency.^13^, ^15^, ^16^, ^27^ However, formation of CO as it has been reported as alternative side reaction besides hydrogen evolution^13^ cannot be excluded, but has not been examined.

The achieved FE for formate generation is lower compared to previously stated values from literature. Exemplarily, Kopljar et al. reported a FE around 90 % with current densities up to −200 mA cm^−2^ using 0.1 M KHCO_3_ (pH=10) in a semi batch process and 1 cm^2^ of a Sn doped GDE.^13^ Based on these results Kopljar further described a FE for formate synthesis of 75 % with current densities as high as −400 mA cm^−2^ with alkaline KHCO_3_ in a continuous reactor setup.^15^ Furthermore, De Mot et al reported the electroreduction of CO_2_ to formate in a sophisticated reactor with an electrode surface of 16 cm^2^. Thereby, a current density of −100 mA cm^−2^ yielded a FE of 75 % was observed in 0.5 M KHCO_3_.^17^ Consequently, increasing FE of the described formate electrosynthesis within this study should be the aim of further research to improve the overall performance of the process. The different mechanistic approaches for the electrochemical CO_2_ reduction have been intensively reviewed elsewhere,^1^, ^28^, ^29^ and were not within the focus of this study. Measures to further increase FE might range from optimization of the GDE to electrolyte composition (such as higher pH value^13^ or higher electrolyte concentration^17^) via electrolysis conditions (especially gas and fluid pressure at the GDE) to the reactor set‐up.^17^, ^30^, ^31^ However, measures such as a significant increase of electrolyte concentration or pH value are contradictory to physiological conditions for microbial product formation. Increasing the availability of CO_2_ for the galvanostatic process should allow a higher conversion of CO_2_ and thereby supress hydrogen formation as the main side reaction.

However, the presented scalable approach provided the synthesis of formate in a physiological buffer in terms of pH, salinity and substrate concentration, which was directly used for biotransformation.

### Cultivation of *C. necator* on formate medium

Prior to the bioconversion of formate of electrochemical origin to PHB, *C. necator* was grown on 100 mm formate medium to adapt cells to the use formate as sole carbon and energy source. The cultivation was conducted in a medium modified from Sydow et al., by an exchange of the growth substrate and an increase in buffer capacity.^32^ The growth curve including the pattern of the formate concentrations has been deposited in the Supporting Information (Figure S2). Briefly, the maximum growth rate was 0.23±0.05 h^−1^ and the maximum substrate consumption rate was 8.6±1.82 mmol L^−1^ h^−1^. The highest OD_600_ of 1.1±0.4 was observed after 21.75 h and decreased to 0.7±0.1 at the end of cultivation. Complete formate consumption was observed after 40 h. As already mentioned before, the main growth limiting factor for *C. necator* under the presented conditions besides substrate availability was the regulation of the pH value, which is related to the organism's ability to consume the carboxylic acid anion formate. Within this study the pH value was regulated manually with H_2_SO_4_, since the aim was to keep change in media composition minimal and prevent pH‐induced influences on the formate amount in the shaking flasks. For future transfer of the cultivation to larger scale, the approach of combining feed and pH regulation by the addition of formic acid in a continuous bioreactor system a rather favourable since the substrate depletion and pH regulation can both be controlled by the addition of formic acid.^33^

### Formate transformation to PHB

Based on the growth on formate medium, the PHB production by *C. necator* resting cells from formate was subsequently examined in two steps: initially, PHB was synthesised from formate of non‐electrochemical origin in formate medium and finally from formate in electrolysis buffer.

**Figure 2:**
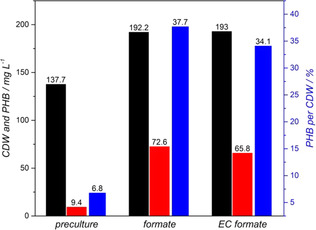
Comparison of CDW (black columns), PHB concentration (red columns) and PHB per CDW ratio (blue columns) of *C. necator* incubated on 100 mm formate medium (*preculture*), 100 mm formate medium lacking (NH_4_)_2_SO_4_ (*formate*) and 93.5±2.0 mm formate containing electrolysis buffer (*EC formate*). *n*=2 for preculture, *n*=1 for *formate* and *EC formate*.

For the PHB production on formate of non‐electrochemical origin, formate pre‐grown resting cells were added to 100 mm formate medium lacking ammonium to prevent growth and to direct formate consumption to intracellular PHB formation. Time course of both formate concentration and OD_600_ over 28 h are presented in Supporting Information (Figure S3). Briefly, 100 mm formate were continuously consumed by the resting cells, with an average consumption rate of 3.2±0.1 mmol L^−1^ h^−1^ (*n*=3) and a remaining formate concentration of 4.5±1.8 mm (*n*=3). The OD_600_ was continuously increased from the starting value of 0.5±0.1 (*n*=3) to a final value of 1.6±0.1 (*n*=3) after 28 h of incubation. Since the medium was lacking ammonium and cells were washed prior to PHB production, growth of *C. necator* was excluded. The OD_600_ increase can be related to the intracellular PHB production and an increase of cell size caused by the intracellular PHB granules, as it has been reported in literature before.^34^ Results of the CDW determination as well as the PHB content referred to CDW are presented in Figure 2. After 28 h incubation of *C. necator* resting cells in 100 mm formate medium without ammonium, a final concentration of 192 mg CDW L^−1^ was measured. GC analysis of the lyophilized cell pellet gave a final PHB concentration of 73 mg L^−1^, resulting in a ratio of 38 % PHB per CDW. Considering that pre‐culture *C. necator* cells already contained intracellular PHB after 24 h of growth originating from the 100 mm medium, the CDW and PHB concentration are also presented in Figure 2. As can be seen, even before incubation under ammonium limitation cells already started PHB production, resulting in a 9 mg L^−1^ PHB concentration (7 % g PHB g^−1^ CDW^−1^) after 24 h pre‐cultivation. Addressing the obtained results for the optical density (OD_600_) and CDW, it must be mentioned that the authors were not able to reasonable correlate OD_600_ values and CDW with PHB producing *C. necator* cells.

Compared to literature, a PHB content of 38 % for *C. necator* is relatively low. Values from 70 up to almost 90 % PHB per CDW have been reported employing different substrates.^34^, ^35^, ^36^, ^37^ However, the pattern of formate concentration leads to the assumption that under the given conditions mainly the depletion of formate limits further PHB production. It is assumed that under a constant substrate feed significantly higher PHB per CDW rations are feasible.

**Figure 3:**
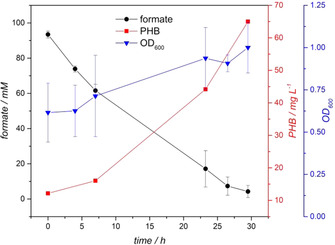
Time course of formate concentration, PHB concentration and optical density (OD_600_) observed for the incubation of *C. necator* resting cells on 0.2 M phosphate electrolysis buffer containing 93.5±2.0 mm electrochemically synthesised formate. Mean±SD, *n*_initial_=9, *n*_final_=3.

Within the next part of this study, *C. necator* resting cells were used to produce PHB from formate, which was previously synthesised by electrochemical CO_2_ reduction. Importantly, electrosynthesis and microbial substrate conversion were performed within the same buffer (0.2 M phosphate) without any auxiliary treatment of the electrolysis buffer between both processes. Similar to medium based growth on formate and PHB production the regulation of the pH value was a crucial factor for the PHB production based on electrochemically generated formate as well. The results for development of the optical density (OD_600_) and formate concentration as well as the pattern of PHB concentration are presented in Figure 3. The OD_600_ was constantly increased from the starting value 0.6±0.2 (*n*=9) to a final value of 1.0±0.2 (*n*=3) after 29.5 h of incubation. The OD_600_ pattern was comparable to the observations made for resting cells in the ammonium free formate medium, but in numbers the OD_600_ increase was smaller for the resting cells incubated on the electrolysis buffer. The formate concentration was decreased with an average consumption rate of 3.0±0.1 mmol L^−1^ h^−1^ from the starting concentration of 93.5±2.0 mm (*n*=9) to a final remaining concentration of 4.2±3.5 mm (n=3) after 29.5 h. For the production of PHB from formate, three triplicates were run in parallel, allowing the observation of the PHB production over time, which is presented in Figure 3. Initially, 9 mg L^−1^ originating from the pre‐cultivation are present as starting concentration. With increasing incubation time the PHB concentration was increased to a final value of 66 mg L^−1^, resulting in the ratio that 34 % of the CDW are PHB (Figure 2). It has to be highlighted, that the final CDW, PHB concentration and ratio of PHB to CDW as well as formate consumption rates of both PHB production on non‐ and electrochemical formate show very similar results. The formate containing electrolysis buffer could directly be used for the subsequent biotransformation without any further processing steps (pH adjustment, dilution, removal of side products etc.). These results indicate that under the given experimental conditions, PHB production with *C. necator* directly on the formate within electrolysis buffer is straight forward and a direct coupling of the two processes is possible.

Subtracting the PHB from precultivation, a final concentration of 56 mg L^−1^ PHB was synthesised from electrochemically produced formate. This value is higher compared to previously reported results reported by Al Rowaihi et al. (25.2 mg L^−1^).^23^ Furthermore, it has to be mentioned that contrary to the results reported by Al Rowaihi et al., within this study the conversion of formate to PHB could also be shown without the application of electrolysis and the consequently assumed presence of reactive oxygen species.^23^

However, when evaluating the PHB concentration, the starting biomass concentration and substrate availability also have to be taken into account. It is assumed that with a higher amount of resting cells and constant substrate feed significantly higher product concentrations are feasible. Therefore, this study aims towards the continuous coupling of electrochemical substrate synthesis and direct feed in a sophisticated bioreactor system as it has nicely been demonstrated by Haas et al. with the CO_2_ electrolysis to syngas and its fermentation to alcohols by *Clostridia*.^10^

As final part of this study, the FE of the whole process ranging from electrochemical CO_2_ fixation to the biological PHB production was calculated. Therefore, the amount of carbon, which was incorporated in PHB was related to the amount of consumed formate and the FE of the electrolysis. Based on the GC analysis of the monomer unit 3‐hydroxybuturic acid, 7.1 mm carbon was fixed as PHB, representing 8 % of the totally consumed formate (89.2 mm). Combining this with the FE of the CO_2_ electrolysis, the FE of the overall process is as high as 4 %. When discussing this FE, that the main potential of the process naturally lies in improving the biological substrate conversion. To the author's opinion, two different approaches appear promising: on the one hand, a biotransformation in a bioreactor system, providing ideal fermentation conditions such as constant feed and automated pH regulation, might improve the substrate to product conversion. On the other hand, the rather inefficient Calvin cycle, on which the PHB production is based on might be replaced by a more efficient metabolic cycles such as the reductive glycine pathway, as it has been recently demonstrated by Claassens et al.^38^

Nevertheless, the molecular complexity of PHB as model polymer must be taken into account for the evaluation of the process. Comparing with literature, Krieg et al. reached a FE of 0.92 % for the microbial electrosynthesis of the complex molecule α‐Humulen from CO_2_.^24^

## Conclusions

The aim of this study was to establish the scalable coupling of electrochemical formate synthesis from CO_2_ and the production of a complex bioproduct by a spatial separation of both processes by the development of sustainable drop‐in electrolysis. Therefore, formate was synthesised employing a Sn‐based gas‐diffusion electrode (GDE) in a physiological electrolysis buffer in a custom designed reactor and subsequently transformed to the model product polyhydroxybutyrate (PHB) by *C. necator*. With this study, it was demonstrated that a coupling by a spatial separation of both processes is feasible without the application of further downstream processes between substrate generation and product formation. Furthermore, by a spatial separation the requirements of both the electrochemical CO_2_ reduction (e. g., current density, minimum electrode spacing) and biotechnological transformation (e. g., constant substrate feed, pH regulation) can be faced with less compromises such as non‐ideal electrolysis reactor design compared to the in situ generation of the electrochemical intermediates. However, it must be stated that the performance of each individual process step has to be improved to reach a higher faradaic efficiency (FE) for the overall process. According to our calculations on the overall FE, the optimization of the microbial substrate conversion offers a promising potential towards high process efficiencies.

With this work, we present an industrially relevant and scalable combination of two processes merging the sustainable utilisation of CO_2_ as resource, the storage of CO_2_ and energy as an uncritical and easy to handle electrochemical intermediate and the biological transformation to the higher value model compound PHB.

## Experimental Section

### Electrochemical formate synthesis from CO_2_

#### Reactor setup

**Figure 4:**
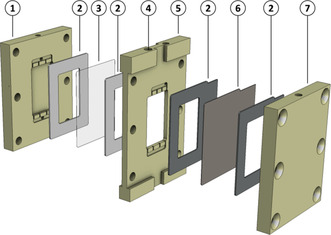
Schematic illustration of the custom designed electrolysis reactor composed of the following parts: anode chamber with spatial cut‐outs (1), silicon gaskets (2), membrane (3), RHE port (4), cathode chamber with spatial cut‐outs (5), GDE (6), gas chamber (7). A detailed description can be found in the main text.

The formate electrosynthesis was performed in a newly custom designed reactor made from PEEK (polyetheretherketone). The detailed layout is presented in Figure 4. It incorporated three compartments, one for gaseous CO_2_, the catholyte and the anolyte each. The gas chamber (2.5 cm×4 cm×0.6 cm) was separated from catholyte chamber by the GDE, which had an accessible geometrical surface of 10 cm^2^ (2.5 cm×4 cm). The GDE was secured between two silicone gaskets (1 mm) to prevent fluid leakage. The catholyte chamber frame separating the GDE and the membrane was 3 mm thick. A proton exchange membrane (2.5 cm×4 cm, Nafion™, PFSA 117, DuPont, Wilmington, USA) divided catholyte and anolyte chamber to enable ion exchange but prevent formate crossover. The membrane was sealed by two silicone gaskets (1 mm). In the anolyte chamber (2.5 cm×4 cm×0.3 cm) a platinated titanium grid (2.5 cm×4 cm) served as anode for the oxidation of water. All three chambers had an in‐ and outlet that were located on the top and bottom of the reactor, each equipped with PP (polypropylene) adapters to connect to CO_2_, catholyte or anolyte supply via Norprene tubes (inner diameter=1.6 mm, Saint‐Gobain, Courbevoie, France), respectively. The catholyte chamber had an additional inlet, in which a reverse hydrogen electrode (RHE, Mini HydroFlex, Gaskatel GmbH, Kassel, Germany) was placed. Inside the reactor in‐ and outflow for catholyte and anolyte had special cut‐outs to guarantee equal flow distribution and electrolyte supply. CO_2_ flowed through the gas compartment top down; the overpressure was adjusted downstream by a needle valve and a pressure sensor (Greisinger, Regenstauf, Germany). Catholyte and anolyte had an individual reservoir each and were circulated employing a peristaltic pump (ECOLINE VC–MS/CA8‐6, ISMATEC, Wertheim, Germany). Moreover, the electrolytes were passed through the reactor bottom‐up to prevent gas entrapment and maintain fluid coverage of the electrodes. During an electrosynthesis current density was controlled and kept at a constant value by a potentiostat, either a Reference 600+ (Gamry Instruments, Pennsylvania, USA) or an Autolab PGSTAT302N (Metrohm, Herisau, Switzerland) were employed. A photograph of the reactor setup and a schematic representation are provided in the Supporting Information (Figure S4).

#### CO_2_ electrolysis

Prototype GDE were kindly provided by Gaskatel GmbH (Kassel, Germany), containing 90 % Sn powder, 5 % PTFE and 5 % activated carbon and were fabricated by calendaring. An incorporated stainless steel mesh served as current collector and a PTFE foil was applied at the gaseous site of the GDE. 100 mL of 0.2 M KH_2_PO_4_/K_2_HPO_4_ (equimolar, pH=6.7) were used as anolyte as well as catholyte and circulated with a flow rate of 80 mL min^−1^ between the reservoirs and the electrode chambers. CO_2_ was flushed through the gas chamber with a flow rate of 30–40 mL min^−1^ and an overpressure set to 10±2 mbar at the gas outlet of the gas chamber. Electrolyses were performed under an incubation hood at 30 °C. The electrochemical CO_2_ reduction to formate was performed at a current density of −50 mA cm^−2^. Initially, current was increased with 1 mA s^−1^ to reach −50 mA cm^−2^ and from then on kept constant until the end of formate synthesis. The resulting electrode potential vs. the RHE was constantly monitored and not IR compensated. In order to evaluate the formate production rate from CO_2_ over time, catholyte samples (1 mL) were taken in intervals of 1 h. The electrosynthesis were run for approximately 2 h to reach a formate concentration of 93.5±2.0 mm for the subsequent biological transformation. The generated formate containing electrolysis buffer was then either directly used for biological transformation or stored at −20 °C.

#### Microorganisms

To demonstrate the bioconversion of the electrochemical intermediate formate *Cupriavidus necator* wildtype (DSM‐428, DSMZ, Braunschweig, Germany) was used as model organism, which produces PHB from formate under nitrogen limitation.

#### Growth media

For the cultivation of *C. necator* the following growth media were used: **Luria Broth (LB)**: 5 g L^−1^ yeast extract, 10 g L^−1^ triptone and 5 g L^−1^ NaCl in deionized water, pH was set to 7.0 with NaOH and HCl. **Formate Medium**: the 100 mm formate medium was derived from the medium described by Sydow et al.^32^ The buffer capacity was increased compared to the original due to a significant pH increase during cultivation caused by formate consumption. The media's composition was the following: 6.8 g L^−1^ NaCOOH; 4.68 g L^−1^ NaH_2_PO_4_; 6.5 g L^−1^ Na_2_HPO_4_; 0.8 g L^−1^ MgSO_4_ ⋅ 7 H_2_O; 0.943 g L^−1^ (NH_4_)_2_SO_4_; 0.016 g L^−1^ FeSO_4_; 1 mL ^−1^ trace element solution (in 0.1 M HCl: 1.5 mg L^−1^ FeSO_4_ ⋅ 7 H_2_O; 2.4 mg L^−1^ MnSO_4_ ⋅ H_2_O; 2,4 mg L^−1^ ZnSO_4_ ⋅ 7 H_2_O; 0.48 mg L^−1^ CuSO_4_ ⋅ 5 H_2_O; 1.8 mg L^−1^ Na_2_MoO_4_ ⋅ 2 H_2_O; 1.5 mg L^−1^ Ni_2_SO_4_ ⋅ 6 H_2_O; 4.02 mg L^−1^ CoSO_4_ ⋅ 7 H_2_O). All media components were prepared sterile in separate stock solutions and combined prior to each experiment. The pH value was set to 6.8 with sterile H_2_SO_4_ and NaOH.

#### Cultivation of *C. necator*

All cultivations were carried out at 30 °C employing a shaking frequency of 180 rpm (shaking diameter of 25 mm). A *C. necator* pre‐culture was raised from a cryo stock in 5 mL LB. After 24 h of incubation, cells were harvested by centrifugation (5 min, 14100 g), washed with fresh formate medium and added to 25 mL of the 100 mm formate medium to reach a starting OD_600_ of 0.05. During the cultivation the pH was constantly measured and manually kept around 7 by the addition of sterile 2 M H_2_SO_4_. To monitor the growth of *C. necator* on the modified formate medium both the optical density (OD_600_) and the formate concentration were measured in intervals over 67 h. Cultivations were run in triplicates.

#### PHB production with *C. necator* from formate

Initially, PHB was synthesized with *C. necator* resting cells from formate of non‐electrochemical origin. Therefore, cells were grown on formate as described above on 100 mm formate medium for 24 h and harvested by centrifugation (20 min, 5000 g) at the end of exponential growth phase. Subsequently, cells were added to 25 mL formate medium lacking (NH_4_)_2_SO_4_ to inhibit growth and thereby initialize PHB production with a starting OD_600_ of 0.5. Optical density and formate concentration were periodically monitored and the pH was manually kept around 7 with sterile 2 M H_2_SO_4_. The cells were harvested for determination of the intercellular PHB content after 28 h. These experiments were performed in triplicates.

To transform the electrochemical synthesized formate into PHB, *C. necator* was grown on 100 mm formate medium to the exponential phase as described before for non‐electrochemical formate transformation. Subsequently, *C. necator* cells were harvested (20 min, 5000 g) and transferred to shaking flasks with 25 mL unsterile electrolysis buffer containing 93.5±2.0 mm formate originating from electrochemical CO_2_ reduction. Cell density was set to a starting OD_600_ of 0.6±0.2 and monitored periodically as well as the corresponding formate concentration. The pH value was manually kept around 7 with sterile 2 M H_2_SO_4_. To monitor the evolution of the PHB concentration during incubation on electrochemically produced formate, initially three triplicates (*n*=9) were cultivated in parallel. After 7, 24 and 29.5 h, respectively, one triplicate was harvested to determine intercellular PHB concentration, resulting in *n*=3 at the end of the experiment.

The PHB content of the preculture was subtracted from the PHB content of the cultures producing PHB from formate under NH_4_^+^ limiting conditions.

### Analytics

#### Formate analysis

The formate concentration in both electrolysis buffer and growth media was analysed by HPLC (Shimadzu) employing the following parameters: Column: Rezex‐ROA, 300×7.8 mm; Phenomenex, California, USA; Method: 5 mm H_2_SO_4_, 0.6 mL min^−1^, 30 °C, 25 min, wave length detector (*λ*=209 nm). As standard for calibration sodium formate solutions were used in the range of 0–220 mm. Samples from cell cultivations were centrifuged (5 min, 14100 g) beforehand to remove planktonic cells.

#### PHB analysis

The PHB concentration of the biomass was determined by acidic methanolysis described by Juengert et al.^39^ Initially, the harvested cells (20 min, 5000 g) were washed with distilled water. The residual cell pellet was stored at −20 °C for at least 2 h. Subsequently, the cell pellet was dispersed in distilled water again and transferred into pre‐weighted pressure tubes (4 mL Ace Pressure Tubes, Sigma Aldrich, St. Louis, USA) and freeze dried for 48 h. The cell dry weight (CDW) of harvested cells was determined by weighing the pressure tubes containing the freeze‐dried cell pellet. 1 mL of chloroform and 1 mL of methanol containing 15 % H_2_SO_4_ were added to the dried cell pellet and heated to 100 °C for 2.5 h before cooling down to room temperature. Subsequently, 1 mL of distilled water and 1 mL of internal standard (2 μL methyl benzoate in 998 μL chloroform) were added to the tubes. After vortexing vigorously for 30 s and a clear phase separation, a sample of the bottom chloroform phase was taken for the GC (Agilent) analysis with the following parameters: Column: DB‐WAXTR 30 m×0,25 μm; J&W Scientific, California, USA; Method: injector to 240 °C, column oven temperature profile: hold at 50 °C for 1 min, increase to 240 °C with 15 °C min^−1^, hold at 240 °C for 5 min; detector: FID. A calibration curve was generated with solutions prepared from commercially available poly‐(3‐hydroxybutyrate) (natural origin, Sigma Aldrich, St. Louis, USA) in the range of 1–50 mg. All pipetting steps involving chloroform were performed with glass syringes and stainless‐steel cannulas.

#### Calculations

The Faradaic efficiency (FE) of the formate synthesis was calculated by following formula [Eq. (1)]:

(1) $${FE}_{{\mathrm{CO}}_{2}\;\mathrm{electrolysis}}=\frac{F\cdot z\cdot n}{I\cdot t}\cdot 100\;\%$$

With: *F*=Faraday constant; *z*=number of transferred electrons (2); *n*=amount of produced formate [mol]; *I*=current [A]; *t*=running time of the electrolysis at constant current density [s].

To evaluate the overall FE from electrochemical CO_2_ fixation to final PHB production the following assumptions were made: the molar amount of produced PHB on formate (electrochemical origin) was determined and referred to the molar amount of consumed formate. This value was then multiplied with the FE of CO_2_ electrolysis to obtain the overall FE for PHB production.

More detailed, the following steps were carried out: since PHB was destructed into single monomer units by acidic methanolysis, the GC signal of the applied PHB standards was referred to the GC signal of 3‐hydroxybuturic acid (factor 2.76). Consequently, the determined PHB concentration was multiplied by the factor, divided by the molar mass of 3‐Hydroxybuturic acid and multiplied by 4 (number of carbon atoms in the monomer) to obtain the concentration of carbon atoms fixed as PHB from formate (electrochemical origin). The calculation is presented below [Eq. (2)]:

(2) $$c_{\mathrm{carbon}\;\mathrm{in}\;\mathrm{PHB}}=\frac{c_{\mathrm{PHB}}\;{}^{*}\;2,76}{M_{3\mathrm{HB}}}\;{}^{*}z'$$

With: *c*_carbon in PHB_=concentration of carbon bound in PHB [mol L^−1^]; *c*_PHB_=concentration of PHB [g L^−1^]; *M*_3HB_=molar mass of 3‐hydroxy‐buturic acid [86.09 g mol^−1^]; *z*′=number of carbon atoms in 3‐hydroxy‐buturic acid.

The molar amount of carbon was the related to the molar amount of consumed formate and multiplied by the FE for CO_2_ electrolysis [Eq. (3)]:

(3) $${\mathrm{FE}}_{\mathrm{overall}}=\frac{c_{\mathrm{carbon}\;\mathrm{in}\;\mathrm{PHB}}\;}{c_{\mathrm{consumed}\;\mathrm{formate}}}\;{}^{*}{\mathrm{FE}}_{{\mathrm{CO}}_{2}\;\mathrm{electrolysis}}$$

With: FE_overall_=Faradaic efficiency of the whole process [%]; *c*_cabon in PHB_=concentration of carbon bound in PHB [mol L^−1^]; *c*_consumed formate_=concentration of consumed formate [mol L^−1^].

## Conflict of interest

The authors declare no conflict of interest.

## Supporting information

As a service to our authors and readers, this journal provides supporting information supplied by the authors. Such materials are peer reviewed and may be re‐organized for online delivery, but are not copy‐edited or typeset. Technical support issues arising from supporting information (other than missing files) should be addressed to the authors.

Supplementary
