# Supplementary material for: From CO2 to Bioplastic – Coupling the Electrochemical CO2 Reduction with a Microbial Product Generation by Drop‐in Electrolysis
Source: ChemSusChem. 2020 Jul 29;13(16):4086–93. doi: 10.1002/cssc.202001235 (PMC7496250; doi:10.1002/cssc.202001235)
Supplement: Supplementary file 1 — Supplementary [file CSSC-13-4086-s001.pdf]

ChemSusChem

Supporting Information

From CO_2 to Bioplastic – Coupling the Electrochemical CO_2 Reduction with a Microbial Product Generation by Drop-in Electrolysis

Markus Stöckl, Svenja Harms, Ida Dinges, Steliyana Dimitrova, and Dirk Holtmann*© 2020 The Authors. Published by Wiley-VCH Verlag GmbH & Co. KGaA.
This is an open access article under the terms of the Creative Commons Attribution License, which permits use, distribution and reproduction in any medium, provided the original work is properly cited.

Results and discussion

CO_2 electrolysis to formate for bioconversion

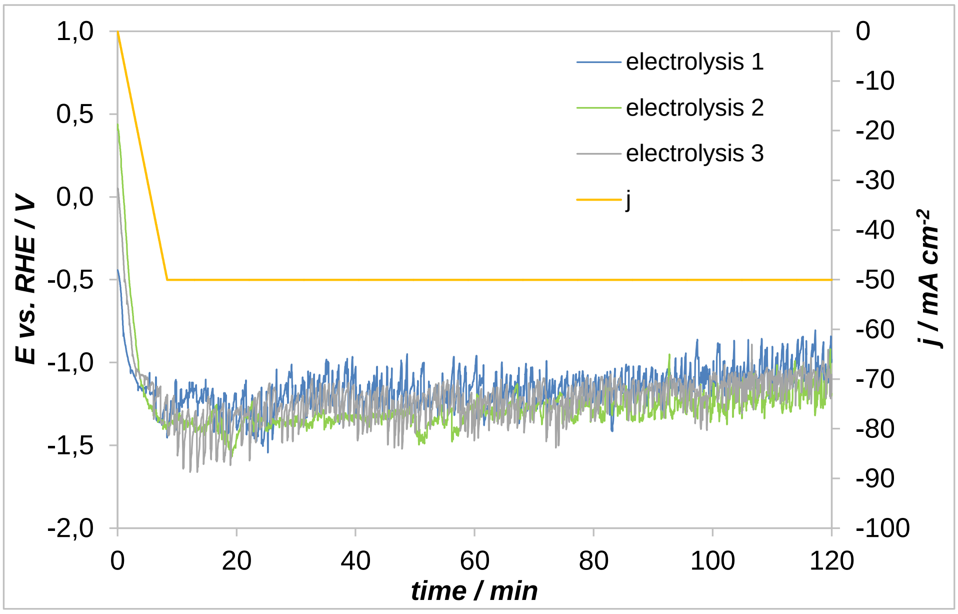

SI figure 1: Time course of electrode potential (*E* vs. RHE, no iR-drop correction) and current density (*j*) during CO_2 electrolysis at Sn-based GDE in a custom designed electrolysis reactor. Blue line: electrolysis 1, green line: electrolysis 2, grey line: electrolysis 3, yellow line: *j*.

Cultivation of *C. necator* on 100 mM formate medium

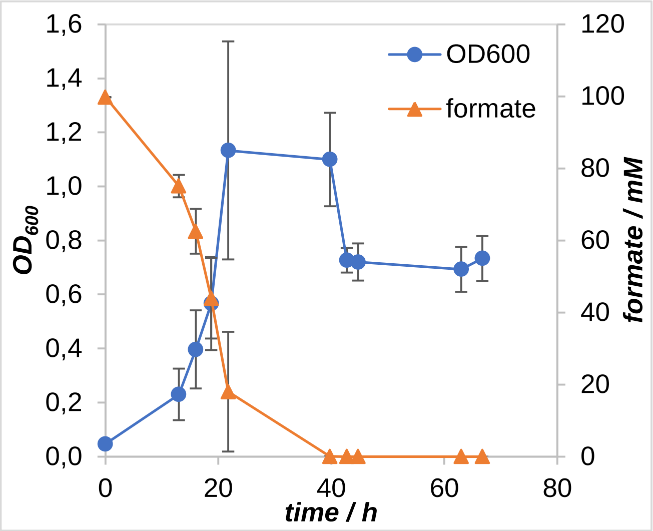

SI figure 2: Time course of optical density and formate concentration observed for the incubation of *C. necator* resting cells on 100 mM formate medium with manual pH control. Blue circles: OD_{600}, orange triangles: formate concentration. Mean ± SD, n = 3.

PHB production with *C. necator* from formate

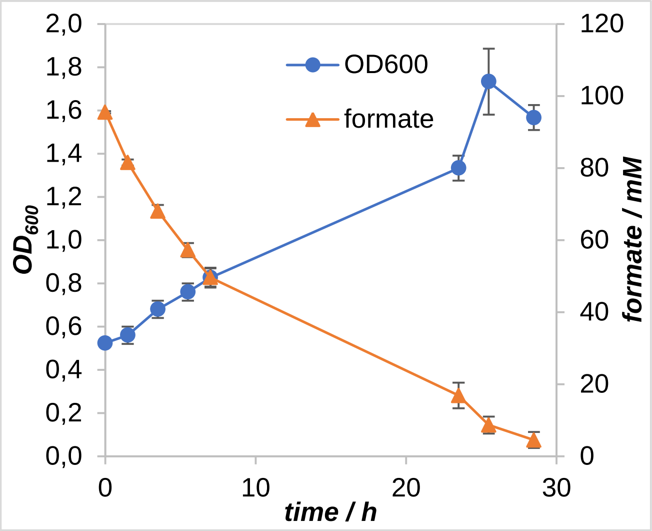

SI figure 3: Time course of optical density (OD_{600}) and formate concentration observed for the incubation of *C. necator* resting cells on 100 mM formate medium lacking $(NH_4)_2SO_4$ with manual pH control. Blue circles: OD_{600}, orange triangles: formate concentration. Mean ± SD, n = 3.

Experimental Section

Electrochemical formate synthesis from CO2

Reactor set-up

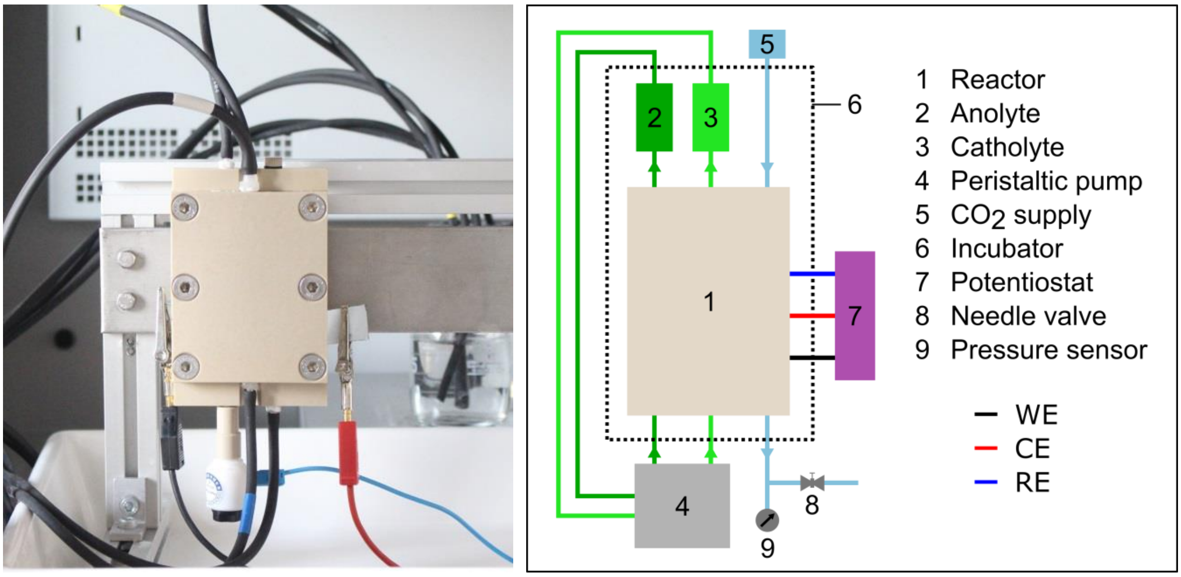

SI Figure 4: Photograph (right) and schematic illustration (left) of the reactor-set-up for CO_2 electrolysis.
